# Sydenham Society

**Published:** 1858-01

**Authors:** 


### EDITORIAL DEPARTMENT.

*Sydenham Society.*—This society, after having published some forty volumes of works which were inaccessible to the English reader, it is feared is about to come to an untimely end. Its members at one time reached some sixteen hundred in number. It has at present nearly a thousand, and is now laboring under financial difficulties. A meeting was to be held on the 7th of November, in London, to devise some measures to prevent the dissolution of the society, but from the tone of the London Lancet, there seems to be but little hope of success. Mismanagement is said to be the cause of its failure. The London Medical Times & Gazette, of Nov. 14th, states that the society is defunct.—*Western Lancet.*

The death of the Sydenham Society will be a calamity indeed, to the profession. The subscription, which was very insignificant, considering the number and quality of books to which it entitled its members, being only five dollars, enabled them to obtain books not elsewhere published in English, and many old works which had gone out of print and which it was exceedingly desirable to revive; and the translations which are so valuable, were entrusted to the most distinguished men in their respective branches, in Great Britain.

We hope that the report of the Medical Times & Gazette is not entirely true, and that the society, though suspended for a time, may rise from its ashes. It is gratifying, however, to the profession to know, that the present difficulties are not attributed to want of appreciation on the part of medical men, but to mismanagement: it seems that with still a thousand members it should be able to hold its own. The corresponding secretary in this country, is Dr. Richard J. Dunglison, of Philadelphia. F.

